# Management and Outcomes of Anti-CD38 Refractory Patients: The Impact of Retreatment and of Subsequent Therapies

**DOI:** 10.1097/HS9.0000000000000975

**Published:** 2023-11-03

**Authors:** Efstathios Kastritis, Foteini Theodorakakou, Ioannis Ntanasis-Stathopoulos, Vassiliki Spiliopoulou, Eirini Solia, Panagiotis Malandrakis, Rodanthi Syrigou, Nikoleta Kokkali, Magdalini Migkou, Evangelos Eleutherakis-Papaiakovou, Despina Fotiou, Maria Roussou, Nikolaos Kanellias, Maria Gavriatopoulou, Evangelos Terpos, Meletios A. Dimopoulos

**Affiliations:** 1 Plasma Cell Dyscrasia Unit, Department of Clinical Therapeutics, National and Kapodistrian University of Athens, Greece

The development of resistance to therapy remains the main barrier to cure myeloma, and most patients develop disease that is refractory to treatments, including proteasome inhibitors (PIs), immunomodulatory drugs (IMiDs) and monoclonal antibodies targeting CD38. CD38-based therapy has become a backbone therapy across all lines,^1^ but, more patients will be refractory to these drugs at early lines and management of relapsed/refractory multiple myeloma (RRMM) that is refractory to anti-CD38 therapies is challenging despite the increasing number of new options (antibody-drug conjugated antibodies, bispecific antibodies^2^ and CAR-T cell therapies^3–5^). In clinical practice, the management of such patients is based either in the use of drugs with new mechanisms of action (if available) or in the retreatment with previously used agents. Previous studies have described the poor prognosis of such patients but may differ from todays’ real-world patients.^6,7^ In addition, the outcomes of RRMM refractory to CD38 may have changed with the use of anti-CD38 containing triplets in earlier lines rather than anti-CD38 monotherapy later. Such data are of clinical relevance since they set the benchmarks for the evaluation of new therapies in this disease setting. Herein, we describe the outcomes of patients who failed anti-CD38-containing therapy and identify potential strategies associated with better outcomes, by analyzing a cohort of patients treated with contemporary regimens.

The analysis included consecutive patients with RRMM who progressed during therapy with a daratumumab- or isatuximab-based regimen (index therapy) and who received further therapy, after progression to index therapy. All patients were treated in a single-center and started anti-CD38 therapy from January 1, 2015 to December 31, 2021. The time at which the patients became refractory to anti-CD38 treatment was set as T_0_ for further time-to-event analyses. Triple-class refractoriness and penta-refractory status were based on previously published definitions.^6^ The analysis was based on data from the prospectively maintained Department’s database and assessed for disease response and progression according to standard institutional protocol, using standard International Myeloma Working Group (IMWG) criteria to evaluate response and progression.^8^ An approval by the Institutional Ethics Committee/Scientific Council was obtained for access to anonymized data, analysis, and publication. This is an observational study and there was no primary hypothesis testing; *P*-values are exploratory. Progression free survival (PFS) for the line of therapy post-CD38 failure was calculated from the date of start of this therapy until progression, death or last follow-up date. Duration of response (DoR) was calculated for responding patients (≥partial response [PR]), from the date of first response (≥PR) to the date of progression, death or last follow up. OS post anti-CD38 failure was defined as the time from the start of subsequent line of therapy until date of death or of last follow up.

The study enrolled 183 consecutive patients who received index anti-CD38 therapy. Their median age was 68 years (range 35–89); Table 1 shows their characteristics. Index anti-CD38 treatment was monotherapy (±dexa) in 50% of patients and in combinations in 50% (with IMiDs, 25% and with PIs, 25%). The median time from initial diagnosis to CD38-based therapy was 43 months (range 0–108) and from diagnosis to anti-CD38 refractoriness was 50 months. The response rate to index anti-CD38-based therapy was 55% (complete response [CR]: 5%, very good partial response [VGPR]: 25%, PR: 25%), and median PFS on index therapy was 6.4 months (5.5 versus 7.4 months for those treated with anti-CD38 ± dexa and anti-CD38 triplets, respectively, HR: 0.862, 95% CI, 0.739-1.004, *P* = 0.055). Post progression to anti-CD38 (median of 3 prior lines), all patients were PI and lenalidomide exposed, 82% were PI refractory, 75% were lenalidomide, and 46% were pomalidomide refractory; 72% were triple-class and 25% penta-refractory. Post anti-CD38 failure treatments included PI-based regimens in 40% (23% carfilzomib-based), 30% pomalidomide-containing, 15% belantamab mafodotin, 5% selinexor ± PI while 23% of the patients received another anti-CD38 containing regimen (Table 1). Among patients that received “single agent” anti-CD38, 40% received a new anti-CD38 combination (45% received Dara-Pd, 35% Dara-Rd, 5% Dara-VCd, 15% Dara-Vd). Among those that received combination anti-CD38 regimen, only 8% received a new anti-CD38 combination.

**Table 1:** Characteristic of the Patients at T_0_ and Relevant PFS and OS After T_0_ With 95% CI

	N = 183	Median PFS (mo)	Median OS (mo)
Age (median/range) Age <65 years/≥65 y	68 (35–89) 42%/58%	5.4 (3–7.7)/7.5 (3.9–11.1)	21.5 (9–33.9)/15.6 (9.9–21.4)
Gender, male/female	53%/47%	6 (4.5–7.4)/6.8 (4.8–8.7)	15.5 (5.3–25)/19.3 (12–26.7)
High/standard risk cytogenetics (in N = 121 patients)	30%/70%	4.6 (2.3–9.2)/6.7 (3.7–9.7)	12.8 (5.6–23.7)/19.3 (8.7–29.9)
LDH > ULN/<ULN	44%/56%	5 (2.8–7.2)/9.2 (6.6–11.8)	13.3 (6.5–26.2)/24.5 (16.6–32.5)
Hemoglobin <10/≥10 g/dL	28%/62%	5.1 (3–7.3)/7.4 (5.6–9.1)	6.5 (3.1–10)/24.2 (19.4–29)
Platelets <100/≥ 100 × 10^9^/L	16%/84%	3.2 (2–7.1)/7.4 (5.6–9.1)	5.1 (3.7–6.5)/24.2 (19.2–26.1)
Albumin < 3.5/≥3.5 g/dL	21%/79%	3.4 (1.2–5.7)/7.5 (5.3–9.7)	6 (1.5–10)/24.5 (20.5–28.6)
Lymphopenia <1000/≥1000/µL	36%/64%	5.4 (4.8–6.1)/8.2 (5.1–11.4)	10.3 (5.5–15.1)/24 (19.2–30.2)
eGFR <30 mL/min/1.73 m^2^ eGFR <60/≥60 mL/min/1.73 m^2^	6%/94% 49%/51%	6 (1–18)/6.7 (5.1–8.3) 6 (4.7–7.3)/7.1 (4.8–10.3)	14.5/19.3 16.2 (11.8–26.7)/18.7 (10.1–33.2)
Calcium ≥11/<11 mg/dL	3%/97%	2.2 (1.7–2.7)/6.8 (4.7–8.9)	9 (1–28)/20.2 (13–27.5)
Time from MM diagnosis to anti-CD38 failure	50 mo		
Prior lines of therapy (at T_0_)			
Median	3 (1–11)		
1	6%	11.6 (4.2–19)	NR
2	21.5%	6.3 (4.1–8.4)	21.2 (6.9–36)
3	25.3%	6.4 (3.1–9.5)	19.3 (11.6–26.9)
4	23.4%	5.4 (3.2–7.6)	11.5 (6.3–13.7)
5	18%	7.5 (1.7–13)	21.5 (9.4–41.3)
>5	6%	4 (1–15.8)	13 (3.5–19.2)
1–3 prior lines	53%	7.5 (4.3–10.8)	20.9 (14.1–27.8)
4 or more	47%	5.4 (2.9–8)	12.8 (6.3–19.3)
Duration of index anti-CD38 based therapy (median/range)	7 (1–84) mo		
PFS on index anti-CD38 therapy ≥12 mo/<12 mo	24%/76%	11.3 (6.7–17.9)/5.2 (4.3–6.2)	39 (20–58)/12 (7–17)
Prior response to anti-CD38			
No response	45%	6 (2.7–10.9)	12 (7.3–16.8)
PR	25%	6.4 (1.7–9.2)	24 (13.1–34.8)
VGPR	25%	6.4 (3.9–8.7)	19.2 (9.6–28.9)
CR	5%	6.5 (1.5–19.1)	NR
Prior ASCT, yes/no	38%/62%	3.9 (2–5.8)/7.5 (5.1–10)	21 (10–31.8)/16.6 (8.9–24.3)
Post anti-CD38 status			
Refractory to any PI	82%	6.4 (4.8–7.9)	19.3 (13–25.5)
Carfilzomib refractory	28%	6.8 (2.9–10)	12.6 (6.7–18.5)
Lenalidomide refractory	75%	5.6 (4.1–7.1)	19.2 (12.2–26.3)
Pomalidomide refractory	46%	7.5 (4.6–10.4)	12.8 (6.8–18.8)
Refractory to anti-CD38	100%	6.4 (4.7–8)	17.6 (11.7–23.6)
Triple-class refractory	53.6%	6 (3.6–8.4)	13.3 (5.7–21)
Penta-refractory	16.9%	6.4 (5.5–7.4)	22.7 (21.9–23.5)
1–2 class refractory	29.5%	6.8 (3.1–10.5)	26.7 (17.4–34.5)
Anti-CD38-based combinations after index therapy			
Dara/Isa-Pd	8%		
Dara-Rd	8%		
Dara-Vd	3%		
Other CD38 combination	4%		
Post-CD38 failure regimen			
PI-containing	40%	6.4 (4.8–8)	22.9 (19.9–25.9)
Carfilzomib-containing	23%	6.7 (2.6–10.9)	22.7 (20.1–24.6)
Pomalidomide-based	30%	4.5 (2.6–6.5)	20.9 (12.4–29.4)
Anti-CD38-based	23%	4 (1.7–6.1)	16.6 (6.8–29.9)
Belantamab	15%	9.1 (4.3–13.9)	24.5. (17.5–31.5)
Selinexor ± PI	5%	3.7 (1–11.8)	30 (NE)
Triplet	50%	6(4.6–7.3)	17.9 (11.1–27.3)
Doublet/monotherapy	50%	6.8 (3.2–10.5)	17.3 (12.6–29.3)

ASCT = autologous stem cell transplantation; CR = complete response; eGFR = estimated glomerular filtration rate; LDH = serum lactate dehydrogenase; OS = overall survival; PFS = progression free survival; PI = proteasome inhibitor; PR = partial response; ULN = upper limit of normal; VGPR = very good partial response.

A response in the post-anti-CD38 line was recorded in 43% (95% CI, 36%-51%) and median PFS was 6.4 (95% CI, 4.7-8) months. Given the high rates of PI and lenalidomide resistance, only pomalidomide sensitivity was associated with better PFS, which, however, remained poor (median 7.5 [95% CI, 4.6-10.4] versus 5.2 [95% CI, 2.7-6.5] months, HR: 0.594, 95% CI, 0.396-0.892, *P* = 0.007). The median PFS for triple-class and penta-refractory patients was 6 (95% CI, 3.6-8.4) and 6.4 months (95% CI, 5.5-7.3) (Figure 1A). Table 1 shows the median PFS across various groups. Thrombocytopenia (platelet counts <100 × 10/L) (HR: 1.756, 95% CI, 1.066-2.895, *P* = 0.025) and lymphopenia (<1000/µL) (HR: 1.488, 95% CI, 1.001-2.216, *P* = 0.048) were associated with shorter PFS. A PFS ≥12 months during index anti-CD38 therapy was the most important prognostic factor for PFS post-anti-CD38 failure (median PFS of 11.3 (95% CI, 6.7-17.9) versus 5.2 (95% CI, 4.3-6.2) months, HR: 0.434, 95% CI, 0.268-0.704, *P* = 0.001) (Figure 1C and Table 1) and was independent of the number of prior lines, type of treatment or pomalidomide resistance and of other characteristics. Notably, PFS with anti-CD38 combinations was 11.6 (95% CI, 3.2-20.1) months among patients with a PFS ≥12 months during prior anti-CD38 therapy. Median DOR in the post-CD38-failure therapy was 11.8 months (95% CI, 6.6-17.1) and was not significantly different across different therapies or lines of therapy (3 or less versus ≥4), however, the most important factor was a PFS ≥12 months on index CD38-based therapy (median DOR of 26.7 (95% CI, 11.4-42) versus 9.4 months (95% CI, 5.5-13), HR: 0.309, 95% CI, 0.134-0.711, *P* = 0.004). Among patients treated with CD38-containing regimen post CD38 failure, the median DOR was 7.5 months (95% CI, 3.6-11.5).

**Figure 1.**
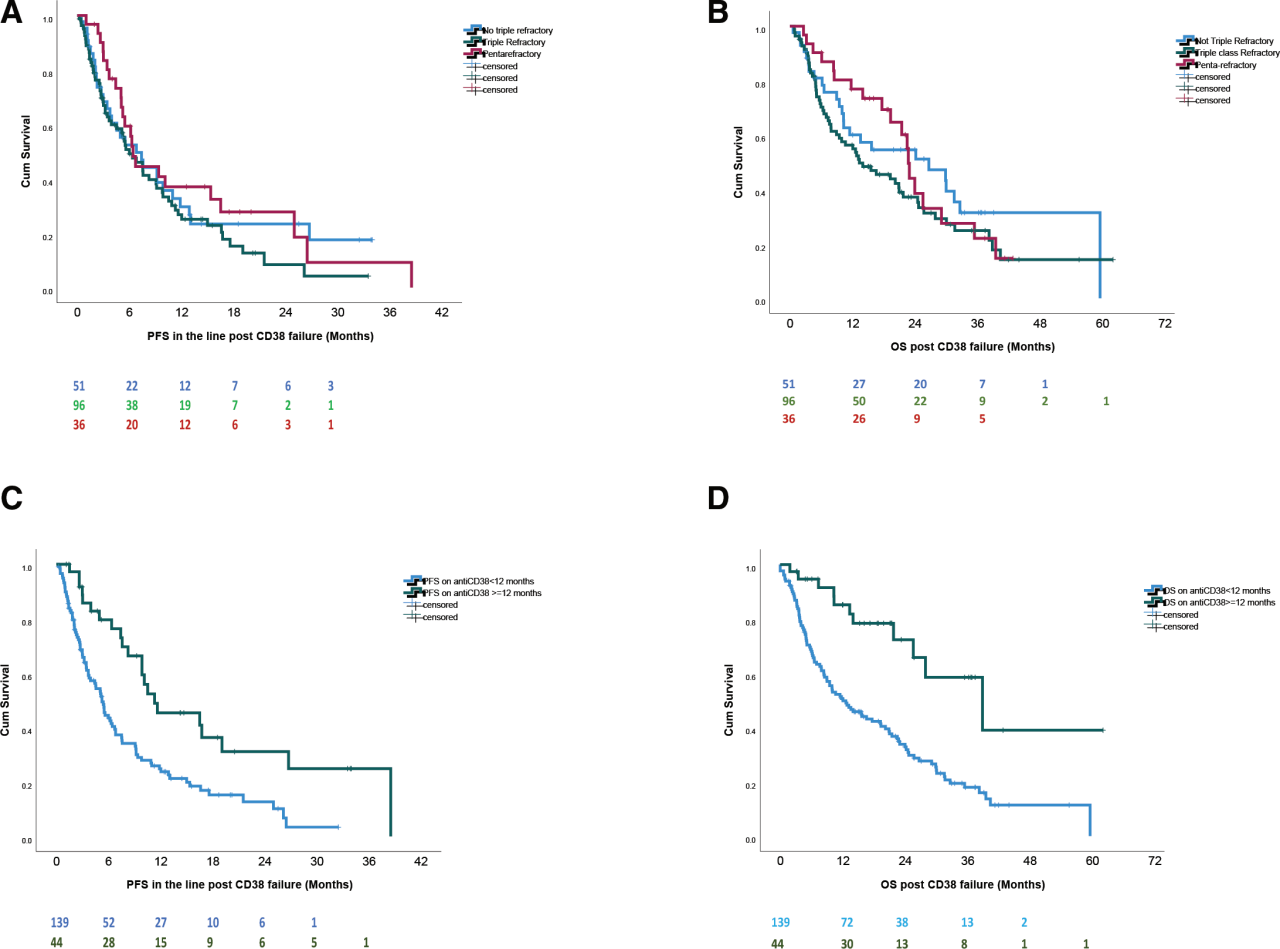
**Outcomes of patients after failure of anti-CD38 therapy.** (A) PFS in the line of therapy post-anti-CD38 failure and (B) OS after anti-CD38 failure, for non-triple-class refractory, triple-class refractory, and penta-refractory patients (C) PFS in the line of therapy post-anti-CD38 failure and (D) OS after anti-CD38 failure for patients who had duration of PFS in the index anti-CD38-based therapy that lasted more or less than 12 months. OS = overall survival; PFS = progression free survival.

The median OS from T_0_ was 17.6 months (95% CI, 11.7-23.6); was 12.8 (95% CI, 7.4-18.2) versus 22.5 (95% CI, 18-27) months for patients who had progressed on antiCD38 ± dexa versus anti-CD38 combinations respectively (HR: 0.910, 95% CI, 0.754-1.099, *P* = 0.327 but patients that received anti-CD38 ± dexa had one additional line of therapy versus those that received triplets (*P* < 0.001); adjusting for lines of therapy, there was no significant difference in the OS (HR: 0.863, 95% CI, 0.577-1.289, *P* = 0.471). Median OS was 13.3 months (95% CI, 5.7-21) for triple-class refractory, 22.7 months (95% CI, 21.9-23.5) for penta-refractory (Figure 1C); in Table 1 the median OS across various groups is shown. The median OS post T_0_ was 39 months (95% CI, 20-58) for those with PFS ≥12 months (versus 12 months (95% CI, 7-17) if <12 months PFS on index anti-CD38 therapy, HR: 0.299, 95% CI, 0.160-0.577, *P* < 0.001) (Figure 1D). Lymphopenia (<1000/μL) (HR: 1.653, 95% CI, 1.093-2.501, *P* = 0.016), thrombocytopenia (platelet counts <100 × 10^9^/L) (HR: 3.79, 95% CI, 2.318-6.199, *P* < 0.001), anemia (hemoglobin <10 g/dL) (HR: 2.338, 95% CI, 1.528-3.577, *P* < 0.001), low serum albumin (<3.5 g/dL) (HR: 3.431, 95% CI, 2.183-5.394, *P* < 0.001) were also associated with shorter OS. In a sensitivity analysis, these characteristics were not associated with the number of prior lines. In multivariate analysis, thrombocytopenia (HR: 2.81, 95% CI, 1.6-5, *P* < 0.001), low serum albumin (HR: 2.49, 95% CI, 1.44-4.3, *P* = 0.001) and a PFS >12 months at index anti-CD38 therapy (HR: 0.367, 95% CI, 0.181-0.745, *P* = 0.006) were the only independent prognostic factors for OS.

The above results confirm that patients failing anti-CD38 therapy have poor prognosis. However, the median DoR was 11.8 months and a group of patients, such as those with previously long-maintained responses to anti-CD38 could achieve quite long DOR (median 26.7 months); this data sets benchmarks for the evaluation of new treatments in this setting and the interpretation of results of recent trials. Although this is not the first study to explore outcomes of patients refractory to anti-CD38-based therapy,^6,7^ it has differences from previously published cohorts and provides a more contemporary view. Compared to the MAMMOTH study^6^ our patients had fewer prior lines (4 versus 3), indicating earlier exposure to anti-CD38 therapy, closer to current real-world paradigm where daratumumab and isatuximab combinations have been approved for use in early lines. All our patients received therapy after T_0_, and 15% received belantamab mafodotin, 5% Selinexor-based regimens, and 23% triplet CD38-containing regimens. Compared to LocoMMotion study,^7^ there are differences in the number of prior lines (4 versus 3), with no significant difference in rates of triple-class and penta-refractory patients (73.8% and 17.7%, respectively) but, in the LocoMMotion study the ORR at the standard of care regimens that were used was 29.8%, the median PFS was 4.6 and the median OS 12.4 months. Our patient population is not significantly different from the KarMMa-3 study cohort (median 3 prior lines), although patients in that study were younger (median 63 versus 68 years), and fitter.^5^ The median PFS in the SoC arm of KarMMa-3 was 4.4 months, with 38% of patients treated with daratumumab-based combinations (versus 23% in our study). Thus, our data could serve as benchmarks for comparisons with emerging therapies, using indirect approaches. However, our data come from a single-center cohort which may hamper definite conclusions, not being representative of other practices.

Recycling previous therapies, in different combinations, is a bridging strategy that offered prolonged remissions only in a minority of patients^9–11^; new treatment approaches and strategies should be prioritized for patients failing anti-CD38. The DoR to prior CD38-targeting therapy was prognostic for the outcomes on subsequent CD38-based combinations therapy; a similar observation for the duration of prior response to IMiD when switching from lenalidomide to pomalidomide has also been reported by our group^12^ and others.^13^ The cutoff of 12 months for the duration of PFS to index anti-CD38, could be cohort-specific and may differ in different cohorts. Since the cutoff date of this study, treatment options have significantly evolved (bispecific antibodies^2^ and chimeric antigen receptor T cells^3–5^), but the evaluation of their impact will need further real-world studies, as they were not available at that time; in the context of availability of these options, our study may have a time bias. Nonetheless, our data in combination with data from the previous studies indicate that refractoriness to anti-CD38-based therapy is a critical point in the natural history of the disease. Notably, there were no significant differences in the PFS post-CD38 failure for those who were triple-class or penta-refractory versus those who were refractory to anti-CD38 plus one more drug. The underlying biology of refractoriness to CD38-targeting therapy is not well understood but may involve both the clone and the immune micro- and macro-environment.

## AUTHOR CONTRIBUTIONS

EK designed the study, collected data, performed the analysis, and wrote the manuscript; FT, IN-S, VS, ES, PM, RS, NK, MM, EE-P, DF, MR, N. Kanellias, and MG collected data and critically reviewed the manuscript; ET and MAD provided data and critically reviewed the manuscript.

## DISCLOSURES

EK: GSK: Honoraria; Genesis: Honoraria; Janssen: Consultancy, Honoraria, Research Funding; Takeda: Honoraria; Amgen: Consultancy, Honoraria, Research Funding; Pfizer: Consultancy, Honoraria, Research Funding. MG: Karyopharm: Consultancy, Honoraria; GSK: Consultancy, Honoraria; Janssen Cilag: Honoraria; Sanofi: Honoraria; Genesis Pharma: Honoraria; Takeda: Consultancy, Honoraria; Amgen: Consultancy, Honoraria. ET: GSK: Honoraria, Research Funding; BMS: Honoraria; EUSA Pharma: Honoraria, Other: Travel expenses; Amgen: Honoraria, Other: Travel expenses, Research Funding; Sanofi: Honoraria, Research Funding; Takeda: Honoraria, Other: Travel expenses, Research Funding; Genesis: Honoraria, Research Funding; Janssen: Honoraria, Research Funding; Novartis: Honoraria. MAD: Jannsen: Honoraria; BeiGene: Honoraria; BMS: Honoraria; TAKEDA: Honoraria; Amgen: Honoraria.

## SOURCES OF FUNDING

The authors declare no sources of funding.
